# Risk Factors of Acute Hand Injuries in Manual Workers: A Case-control study

**DOI:** 10.1038/s41598-017-12385-5

**Published:** 2017-09-22

**Authors:** Hongyi Zhu, Xiaozhong Zhu, Changqing Zhang, Xianyou Zheng

**Affiliations:** Department of Orthopaedic Surgery, Shanghai Jiaotong University Affiliated Sixth People’s Hospital, Shanghai, 200233 China

## Abstract

The purpose of this study was to identify the risk factors of hand injuries in manual workers. Total 1672 manual workers with acute hand injuries who visited our emergency department accompanied by their colleagues from 1 March 2014 to 1 March 2017 were included in this study. One accompanied colleague with identical work task was chosen randomly as control for each patient. The personal characteristics including gender, age, work experience, acute hand injury history, smoking and alcohol consumption were recorded and compared. Finally, we found the history of severe and multiple times of acute hand injuries, high and very high daily alcohol consumption, working experience from one to two years were risk factors for acute hand injuries in manual workers.

## Introduction

Hands are essential organs and their agility and dexterity are vital to our daily lives. However, hands are complex and vulnerable parts which are often exposed to injury. Vessels, nerves, and tendons were in superficial locations under the skin leading to potential severe injuries.

Although many preventive approaches had been adopted, occupational injuries in manual workers still represented a significant part of acute hand injuries, especially the severe ones^1–3^. More than 50% patients receiving digital replantation were manual workers^4,5^. Hence, it is important to investigate the risk factors of acute hand injuries in manual workers for further more effective prevention.

## Methods

**Figure 1:**
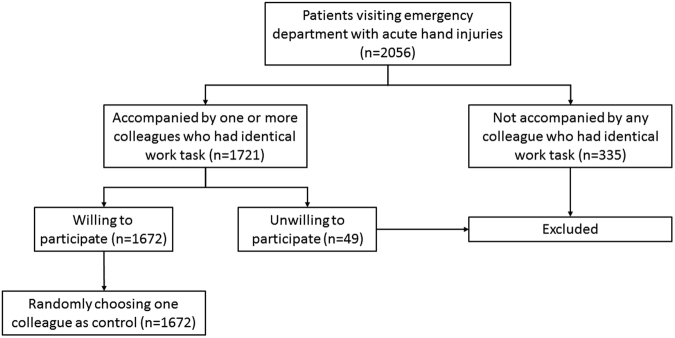
A diagram of participant inclusion and exclusion.

The study was approved by the Ethics Committee of Shanghai Jiaotong University Affiliated Sixth People’s Hospital. Informed consent was obtained from all participants and all methods in this study were in accordance with the Declaration of Helsinki. For the purpose of the study, patients without the accompany of colleague (with identical work task) were excluded. If there were more than one colleagues with identical work task, only one colleague was chosen randomly as control (Fig. 1). The severity of injury was classified into four levels. Level I was defined as the condition without any injuries on bone, tendon, nerve or artery. Level II was defined as single injury on bone, tendon, nerve or artery. Level III was defined as poly-trauma on bones, tendons, nerves or arteries. Level IV was defined as injuries requiring replantation, amputation or flap covering. Daily alcohol consumption was self-reported and categorized as low, moderate, high or very high risk according to the World Health Organization^6^. The cut-offs were “>20”, “>40” and “>60” g/d for women and “>40”, “>60” and “>100” g/d for men, respectively.

All statistics in this study was conducted using SPSS 22.0. The differences were considered significant if the P value was below 0.05. The significance of differences between groups in descriptive and numeric variables were assessed using Pearson’s Chi-squared test and Student’s t test respectively. All data were presented as mean ± standard deviation.

## Results

The statistical analysis of each variable in the two groups were shown in Table 1. Age and gender showed no relevance to the incidence of acute hand injuries. Interestingly, the persons with working experience less than one year did not show a tendency for acute hand injuries (*P* = 0.492). Instead, the persons with working experience from one year to two years were more susceptible to acute hand injuries (*P* = 0.001). High and very high daily alcohol consumption was more frequently observed in the patient group compared with the control group (*P* = 0.031). In contrast, smokers were less susceptible to acute hand injuries (*P* = 0.026). Most importantly, the history of acute hand injuries showed the highest relevance to the incidence of acute hand injuries. The proportion of persons with past level III-IV acute hand injuries and more than two times of past acute hand injuries were both significantly higher in the patient group (*P* < 0.001).

**Table 1:** Comparison of sociodemographic features, medical history findings in study subjects.

	Patient (n = 1672)	Control (n = 1672)	Odds ratio	*P* value
**Gender**				
Male	1373	1385	0.95 (0.80–1.14)	0.585
Female	299	287		
Age	38.5 ± 11.3	38.2 ± 11.7	N/A	0.757
**Work types**				
Manufacturing	869		N/A	N/A
Construction	315			
Other	488			
**History of acute hand injuries**				
Past level III-IV acute hand injuries	201	72	3.04 (2.30–4.01)	<0.001
>2 times of past acute hand injuries	112	23	5.15 (3.27–8.11)	<0.001
**Work experience**				
less than one year	134	145	0.92 (0.72–1.17)	0.492
1–2 years	176	123	1.49 (1.16–1.89)	0.001
High and very high daily alcohol consumption	324	276	1.22 (1.02–1.45)	0.031
Smoking	372	427	0.83 (0.71–0.98)	0.026

Odds ratio are presented as value (95% confidence interval).

## Discussion

To reduce the incidence of acute hand injuries in manual workers, it is important to investigate the risk factors. Due to the diversity of work tasks, it is difficult to find a control for the overall population with acute hand injuries. We therefore focused on the patients accompanied by their colleagues. We chose the colleagues with the identical work task as control. This method eliminate the bias introduced by the different work task. In this study, we showed that the history of level III–IV acute hand injuries, more than two times of past acute hand injuries, high and very high daily alcohol consumption, working experience from one to two years were risk factors for acute hand injuries in manual workers.

Notably, the association between the history of past severe acute hand injuries and the incidence of acute hand injuries was clearly still underestimated according to our results. A substantial amount of patients with severe acute hand injuries were unable or unwilling to continue the career as a manual worker. As a result, the actual risk level of severe acute hand injury history was even higher if the patients continues their careers as a manual worker.

Based on above observations, we recommended that the alcohol consumption for manual workers should be limited to reduce the incidence of acute hand injuries. Although smoking could reduce the incidence of acute hand injuries, the odds ratio was only slightly lower than 1 indicating the preventive effect was mild. In addition, smoking could increase the risks for many cancers and impairment of hand functions^7,8^. We therefore was unable to recommend smoking for manual workers. The prevention of acute hand injuries should be more focused on the populations with high susceptibility revealed by our study. The acute hand injuries were a repeated story and the persons with severe and multiple acute hand injuries were more likely to be affected by acute hand injuries again. Career safety education should be emphasized in these populations.

## Conclusion

The history of level III–IV acute hand injuries, more than two times of past acute hand injuries, high and very high daily alcohol consumption, working experience from one to two years were risk factors for acute hand injuries in manual workers.

## References

[1] Zenke Y (2015). [Examination of the Prevention of Severe Hand Trauma Injury Cases due to Occupational Accidents–An Expert Opinion Gathering Meeting]. Journal of UOEH.

[2] Garg R, Cheung JP, Fung BK, Ip WY (2012). Epidemiology of occupational hand injury in Hong Kong. Hong Kong medical journal=Xianggang yi xue za zhi.

[3] de Abreu LB (1991). [Accident prevention and emergency treatment of traumatic hand injuries in the metropolitan area of Sao Paulo]. AMB: revista da Associacao Medica Brasileira.

[4] Breahna A, Siddiqui A, Fitzgerald O’Connor E, Iwuagwu FC (2016). Replantation of digits: a review of predictive factors for survival. The Journal of hand surgery, European volume.

[5] Zhu, X., Zhu, H., Zhang, C. & Zheng, X. Pre-operative predictive factors for the survival of replanted digits. *International orthopaedics* (2017). 10.1007/s00264-017-3416-3 28161852

[6] Morois, S. *et al*. Daily alcohol consumption and sickness absence in the GAZEL cohort. *European journal of public health* (2017). 10.1093/eurpub/ckx012 28339654

[7] Park S (2014). Attributable fraction of tobacco smoking on cancer using population-based nationwide cancer incidence and mortality data in Korea. BMC cancer.

[8] Dreyfuss, D. Calif, E. & Stahl, S. The Adverse Effects of Smoking on the Hands. *Harefuah* **154**, 327–329, 338 (2015). 26168646

